# Platelet-Rich Plasma Centrifugation Changes Leukocyte Ratios

**DOI:** 10.7759/cureus.14470

**Published:** 2021-04-13

**Authors:** Theodore E Harrison, Jannice Bowler, Todd N Levins, K. Dean Reeves, An-Lin Cheng

**Affiliations:** 1 Pain Management, Private Practice, Sydney, CAN; 2 Pain Management, Private Practice, Victoria, CAN; 3 Integrative/Complimentary Medicine, Private Practice, Victoria, CAN; 4 Rehabilitation Medicine, Private Practice, Kansas City, USA; 5 Biomedical and Health Informatics, University of Missouri Kansas City School of Medicine, Kansas City, USA

**Keywords:** centrifugation, platelets, platelet-rich plasma, leukocytes, leukocyte ratios

## Abstract

Platelet-rich plasma (PRP) is usually described with respect to its platelet concentration and sometimes the concentration of erythrocytes and leukocytes. In this study, we examined the numbers of leukocyte subtypes in PRPs prepared by five different methods. Single spin PRP methods evaluated resulted in a significant increase in the percentage of lymphocytes and proportional/inverse reduction of the percentage of granulocytes in comparison to those percentages found in whole blood. We propose that the centrifugation process traps the denser granulocytes in the RBC layer more readily than lymphocytes and that this will vary by g force and time. The PRP preparation method may be clinically relevant, because the distribution of leukocytes may affect clinical outcomes.

## Introduction

The differences in outcomes of clinical trials with platelet-rich plasma (PRP) have been attributed to variability in the composition of PRP. Several PRP classification systems have been promulgated in order to decrease the confusion surrounding the definition of PRP [1-4]. These systems typically include platelet count, erythrocyte count, leukocyte count and the addition of activating factors to the PRP.

The classification systems have not, however, discriminated between the subtypes of leukocytes. We observed, in a previously published study comparing various methods of PRP preparation for comparative platelet yield, that there appeared to be a difference in the percentage of leukocyte subtypes between whole blood and PRP [5]. Similarly, Carr et al. in 2016 noted a significant change in concentrations of lymphocytes and neutrophils in PRP made from canine blood by five different methods [6] and Baria et al. in 2019 [7] measured an increase in PRP lymphocytes and decrease in neutrophils when preparing PRP in the same system we used for our machine method (although using different settings).

Whether the presence of leukocytes is helpful or harmful remains to be determined, but it may depend on exactly which kinds of leukocytes are present and in what proportions in the PRP. Since leukocyte subtypes differ significantly in their properties [8], an excess of one subtype relative to others may significantly affect the properties of the PRP.

In this study, we investigated the change in percentage of lymphocytes, MIDs (mid cells: eosinophils, basophils, and monocytes combined), and granulocytes found in whole blood complete blood count (CBC) and prepared PRP for five different methods of preparation: machine method (MA), double syringe (DS), single syringe (SS), gel tube (GT), and yellow top tube (YTT). Our hypothesis was that the centrifugation step in PRP preparation would result in a consistent increase in relative lymphocyte percentage, and that this increase would vary significantly between various methods of preparation.

## Materials and methods

Anonymized complete blood counts (CBC) for whole blood and PRP samples prepared from the same subjects’ whole blood were obtained from a previous study of PRP that was reviewed and approved by the ICMS Institutional Review Board: ICMS Approval Number: ICMS-2017-003. That study [5] provides a detailed description of the preparation methods. We summarize each method here:

 *Gel-tube Method* ~10cc of blood was spun at ~950g for ten minutes in a tube containing 1-2cc of a gel with a density slightly higher than platelets. The bottom 3cc of the plasma layer was removed as PRP. *Double-syringe Method* ~15cc of blood in an ACP double syringe (Arthrex ACP Double-Syringe System; Arthrex Inc., Naples, FL, USA) was spun at 300g for five minutes and the plasma layer was withdrawn as PRP.

 *Machine Method*

 90-180cc of blood was placed into an Angel PRP machine (Angel® Concentrated Platelet Rich Plasma System; Arthrex Inc., Naples, Florida, USA) and processed with the setting set for a hematocrit of 4%.

 *Yellow-top Tube Method*

 An ACD-A blood collection tube (BD Vacutainer ACD, catalog #364606; Becton-Dickinson, Franklin Lakes, NJ, USA) was filled with blood and spun at 1000g for ten minutes. The buffy coat and 1-2cc of the plasma layer just above it was extracted for PRP.

 *Single-syringe Method*

 15cc of blood was drawn into a syringe containing 1.5cc of sodium citrate solution and spun for ten minutes at 1000g. 0.6cc just below the buffy coat and 4cc above the buffy coat were removed as PRP.

Red blood cell count (RBC), leukocyte count (WBC), lymphocyte percentage (LYM%), MID cell percentage (MID%) and granulocyte percentage (GRAN%) were recorded from each CBC and PRP preparation for each of the five different methods. Baseline whole blood CBC data were analyzed by an ANOVA with a Bonferroni correction for multiple groups to evaluate for any baseline between-method difference. A Levene test for homogeneity of variance was applied. Change variables were computed for WBC count, RBC count, lymphocyte percentage, MID percentage, and granulocyte percentage for each sample. The presence or absence of a significant between-method difference was determined by the use of a generalized linear measure multivariate analysis with method as the grouping variable. Data analysis was performed using PASW statistics 19 (IBM SPSS Statistic, Armonk, NY) with two-tailed tests and an alpha level of 0.05. 

## Results

**Figure 1:**
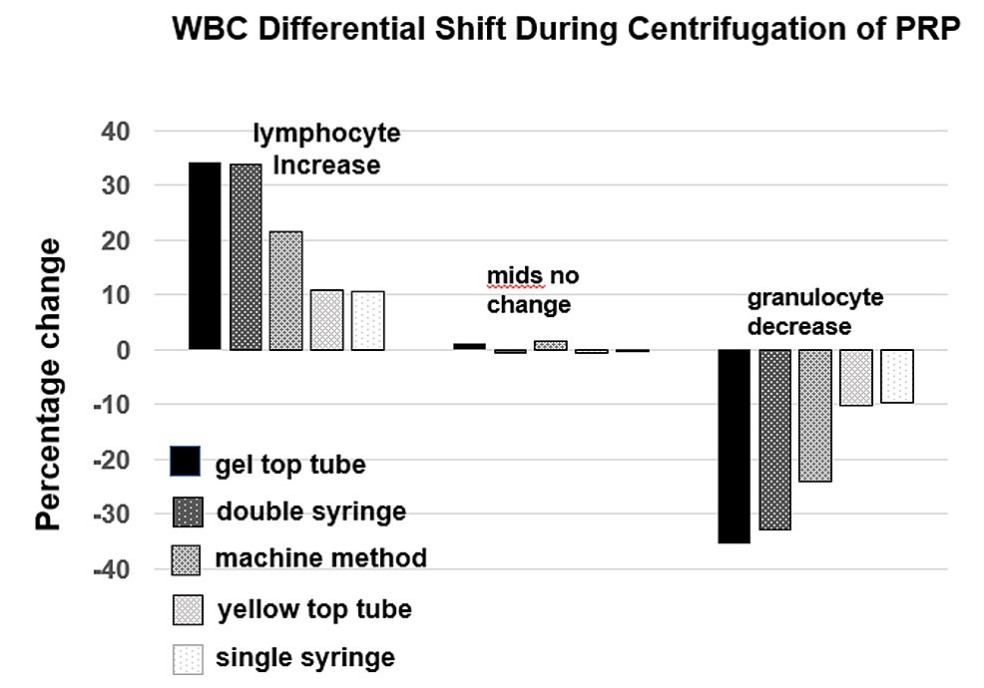
Relative leukocyte subtype shift as a result of centrifugation for PRP preparation for five different PRP preparation methods. Abbreviations: mids: combined eosinophil, basophil and monocyte count; PRP: platelet-rich plasma.

Analysis of baseline whole blood showed no between-method differences for baseline WBC count (p = 0.56), lymphocyte percentage (p = 0.91), MID percentage (p = 0.35) or granulocyte percentage (p = 0.79) (Table 1). However, the RBC count was significantly different between groups (range 4.6 to 5.1; p = 0.003). Including the RBC count as a covariate in analysis, all PRP parameters were significantly different between methods (p < 0.001 for all parameters except MIDs [p = 0.33]) and the change between whole blood and PRP was significant between methods for all parameters measured (p < 0.001) except for MIDs (p = 0.28). Figure 1 shows the changes in percentage of the leukocyte subtypes after centrifugation for PRP.

**Table 1:** Parameters of baseline whole blood and produced PRP and the change between whole blood and PRP values across five methods of preparation. Abbreviations: PRP, platelet-rich plasma; WBC, white blood cell count; RBC, red blood cell count; MID, total eosinophil, basophil and monocyte count.

		WBC	RBC	Lymphocyte percent	MID percent	Granulocyte percent
Gel tube (n = 59)	Whole blood	6.8 (1.9)	4.8 (0.5)	18.5 (5.2)	17.0 (4.4)	63.5 (11.3)
	PRP	4.7 (3.6)	0.1 (0.2)	52.8 (12.8)	18.1 (6.5)	28.2 (12.9)
	Change	-2.1 (4.1)	-4.7 (0.6)	34.4 (12.2)	1.2 (7.8)	-35.3 (18.0)
	Within group, p	<0.001	<0.001	<0.001	0.26	<0.001
Double syringe (n = 32)	Whole blood	7.0 (2.1)	4.6 (0.5)	17.7 (5.6)	16.0 (4.5)	65.8 (8.3)
	PRP	1.8 (1.3)	0.1 (0.2)	51.3 (8.7)	15.3 (4.3)	33.4 (9.8)
	Change	-5.2 (2.6)	-4.5 (0.5)	33.7 (7.4)	-0.6 (5.0)	-32.5 (7.4)
	Within group, p	<0.001	<0.001	<0.001	0.47	<0.001
Machine method (n = 22)	Whole blood	6.3 (1.4)	5.1 (0.5)	18.4 (6.4)	15.1 (3.7)	66.5 (7.9)
	PRP	9.6 (4.8)	0.3 (0.1)	40.0 (14.7)	16.8 (4.1)	42.0 (18.6)
	Change	3.3 (5.0)	-4.8 (0.5)	21.6 (12.0)	1.7 (4.1)	-24.2 (15.4)
	Within group, p	0.006	<0.001	<0.001	0.06	<0.001
Yellow top tube (n = 47)	Whole blood	6.8 (1.9)	4.6 (0.5)	17.7 (5.0)	16.7 (4.4)	64.2 (11.9)
	PRP	16.3 (7.1)	3.3 (1.3)	28.6 (8.6)	16.1 (4.8)	54.3 (12.7)
	Change	9.6 (7.1)	-1.4 (1.3)	10.9 (7.6)	-0.6 (4.0)	-9.9 (13.8)
	Within group, p	<0.001	<0.001	<0.001	0.35	<0.001
Single-syringe (n = 53)	Whole blood	7.1 (2.0)	4.7 (0.6)	18.3 (5.1)	15.6 (4.0)	65.0 (11.3)
	PRP	14.1 (5.3)	1.1 (0.2)	29.0 (8.0)	15.4 (4.4)	55.5 (10.8)
	Change	7.0 (4.2)	-3.6 (0.6)	10.7 (6.0)	-0.2 (4.5)	-9.4 (10.9)
	Within group, p	<0.001	<0.001	<0.001	0.79	<0.001
Between method p-values	Whole blood	0.56	0.003	0.91	0.35	0.79
	PRP	<0.001	<0.001	<0.001	0.033	<0.00
	Change	<0.001	<0.001	<0.001	0.276	<0.001

**Figure 2:**
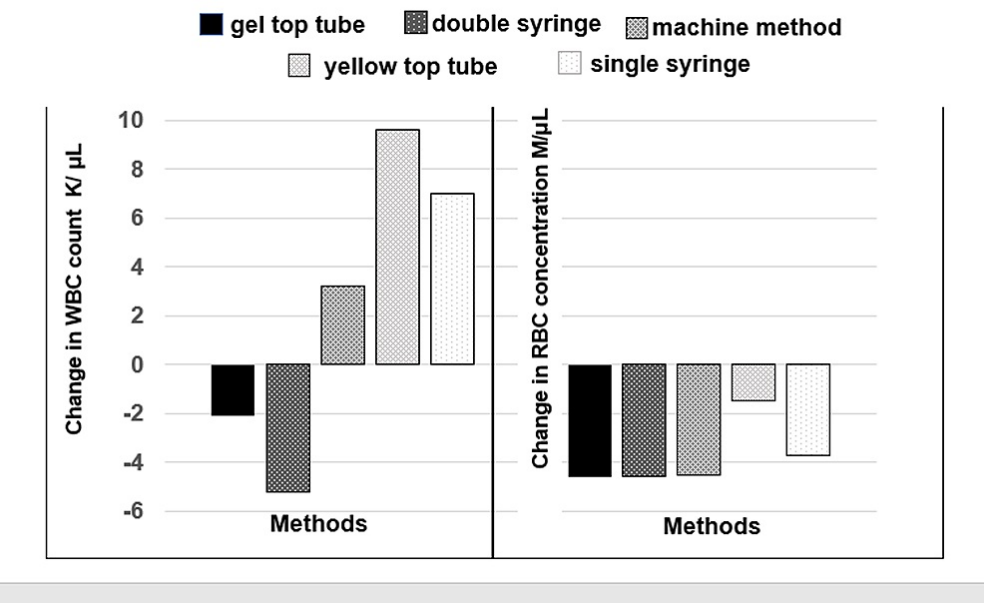
Changes in concentration of leukocytes (WBC) and erythrocytes (RBC) using different methods of PRP preparation. Abbreviations: WBC, white blood cell count; RBC, red blood cell count; PRP, platelet-rich plasma.

There is a significant shift (increase in lymphocyte percentage and decrease in granulocyte percentage) evident across PRP preparation methods (Table 1, Figure 2), ranging from a 10.7% to 34.4% increase in lymphocytes (p < 0.001 for each method) to a 35.3% to 9.4% decrease in granulocytes (p < 0.001 for each method). Pairwise between-group comparisons of estimated marginal means for the percent change in lymphocytes and granulocytes is shown in Table 2, compared to the gel tube preparation method. The increase in lymphocytes and decrease in granulocytes was comparable for gel tube and double-syringe methods (mean difference of only 0.6%; p = 1.0) and significantly more than with the other three methods (machine method [p < 0.001 for lymphocytes and p = 0.016 for granulocytes], yellow-top tube [p < 0.001 for both], and single-syringe [p < 0.001for both]) The percentage change for MIDs between whole blood and PRP did not reach significance for any method (p values range from 0.06 to 0.76) (Table 2).

**Table 2:** Comparison of other methods of PRP preparation to gel tube preparation for relative increase in lymphocyte percentage and decrease in granulocyte percentage in differential counts. ^†^Pairwise comparisons of estimated marginal means for lymphocyte and granulocyte percent change.

Parameter	Method to compare (A)	Other methods (B)^†^	Mean difference (A-B)	Std. error	p-value	95% confidence interval for difference	
						Lower bound	Upper bound
Differences between methods for change in lymphocyte percentage	Gel top tube	Double spin	-0.6	2.1	1.0	-6.5	5.2
		Machine method	-12.8	2.3	<0.001	-19.5	-6.2
		Yellow top tube	-23.4	1.8	<0.001	-28.6	-18.1
		Single-spin	-23.6	1.7	<0.001	-28.6	-18.6
Differences between methods for change in granulocyte percentage	Gel top tube	Double spin	2.5	3.1	1.0	-6.2	11.3
		Machine method	11.2	3.5	.016	1.2	21.2
		Yellow top tube	25.1	2.8	<0.001	17.3	33.0
		Single-spin	25.7	2.78	<0.001	18.1	33.2

Figure 2 shows the change in RBC and WBC after centrifugation. As expected, the RBC count decreased in all preparation methods. Partial correlation analysis of the relationship between the decrease in RBC count and increase in lymphocyte count (controlling for change in WBC count) showed a weak correlation (rp = 0.28). In contrast, partial correlation analysis of the relationship between the decrease in WBC count and increase in lymphocyte count (controlling for change in RBC count) showed a much stronger correlation (rp = 0.51). This confirms that the methods with the greatest decrease in WBC count were the same methods that had the greatest increase in lymphocyte percentage and decrease in granulocyte percentage (Figures 1, 2).

## Discussion

Differential centrifugation is one of the most widely used techniques for the preparation of PRP. It takes advantage of the different densities of the particles in the blood in order to separate them by application of high g-force. Theoretically each particle will migrate to a level in the column of liquid where it is above all the higher density particles and below all the lower density particles. However, this does not happen instantaneously. It takes time for particles to migrate through the sea of other particles to come to their final destination. And while density determines the final resting place of a particle, other factors, such as size, viscosity, and g-force determine how rapidly it gets there.

One of the banes of platelet-rich plasma research has been the wide variety in composition of PRP with regards to not only platelets, but also leukocytes and erythrocytes. Our earlier study of different methods of PRP preparation showed that different preparation methods differed significantly in these parameters [5]. This study shows that the variability is confined not just to the three major cell types but also to the leukocyte cell subtypes as well. Our results suggest that preparation methods that decrease leukocyte count in PRP increase the lymphocyte and decrease the granulocyte fraction of the remaining leukocytes. This may be explained by the higher density of granulocytes relative to lymphocytes. We believe that during centrifugation the denser granulocytes tend to stay trapped in the red cell layer of the centrifugate and thus are not captured in the PRP.

This is significant for PRP prepared by single-spin methods because it shows that at least part of the controversy over whether leukocyte-rich (LRPRP) or leukocyte-poor (LPPRP) PRP [9-11] is clinically better for therapeutic purposes may be due to differences in leukocyte types rather than just leukocyte quantities. Few head-to-head trials have been performed comparing LRPRP and LPPRP, and no definitive conclusions can be drawn at this point. Different clinical conditions may require different preparations [12]. Given the significant differences in leukocyte function, PRP research may be best served by more exactly specifying the type of leukocytes in LRPRP. As more sophisticated technology such as two-spin, three-spin and filtration become more widespread it will be possible to separate leukocytes more precisely in PRP. Since different types of leukocytes differ significantly in function it may be advantageous to produce several different varieties - e.g. monocyte-rich PRP, lymphocyte-rich PRP, neutrophil-rich PRP - for different purposes. It is notable that in this study the relative percentage of MIDs (and by inference, monocytes) did not change in the PRP regardless of the method used.

## Conclusions

This study shows that the single-spin PRP preparation methods that we studied are in fact producing lymphocyte-rich/granulocyte-depleted PRP. We believe these findings will be consistent across all single-spin PRP methods, as all these methods have in common the same centrifugation step that probably traps granulocytes in the red cell layer. The clinical effects of this shift in leukocyte types remain to be seen. Lymphocytes and monocytes may improve healing by their effects on modulation of inflammation, tissue remodeling and repair, and phenotypic display of macrophages. Further investigation is needed to explore the roles of different leukocyte types in PRP.
